# Psychological distress and post-traumatic growth in France during the COVID-19 pandemic: A mediation model of psychosocial safety climate as a determinant of work performance

**DOI:** 10.3389/fpsyg.2022.993458

**Published:** 2022-10-18

**Authors:** Émilie Sandrin, Jean-Pierre Brun, Christophe Nguyen, Caroline Biron, Hans Ivers

**Affiliations:** ^1^Empreinte Humaine, Paris, France; ^2^Department of Management, Faculty of Business and Administration, Université Laval, Quebec, QC, Canada; ^3^VITAM Research Centre on Sustainable Health, Quebec, QC, Canada

**Keywords:** psychosocial safety climate (PSC), psychological distress, post traumatic growth (PTG), performance, pandemic crisis

## Abstract

The psychosocial safety climate (PSC) reflects workers’ perceptions of senior management’s concern for mental health. Because the COVID-19 pandemic has exacerbated organizational issues, PSC could be a target for interventions attempting to preserve both the psychological health of employees and the economic health of companies. This study examines the direct and indirect relationships between PSC and work performance through two indicators of psychological health, psychological distress and post-traumatic growth, during a health crisis, i.e., prior to the second confinement in France. To this end, 2,004 participants from the French workforce completed a survey in October 2020. The results of mediation analyses indicate that PSC has a direct and positive influence on post-traumatic growth (PTG) and performance, as well as a direct negative influence on psychological distress. PSC also has an indirect positive influence on performance via psychological distress. Organizations that wish to jointly address mental health and performance at work would benefit from optimizing PSC.

## Introduction

On March 11, 2020, the World Health Organization (WHO) officially declared the COVID-19 a pandemic (World Health Organization [WHO], 2020), and as we have all seen, the disease rapidly spread across the globe (Bontempi, 2022). The global population has experienced many health restrictions, e.g., lockdowns, curfews, and social distancing, which have required people to adopt new behaviors in all areas of their lives (Raile et al., 2020). In the workplace, the health crisis has led to new organizational practices, such as teleworking (Feng and Savani, 2020), which have greatly transformed employees’ work experiences, e.g., work and home overload while telecommuting (Burk et al., 2021). Some authors underline the pressing need to act to preserve employees’ psychological health during the pandemic (Chen et al., 2021). Long before the pandemic, the WHO already stressed the urgency of increasing investment in mental health because depression was already one of the leading causes of disability in the world (World Health Organization [WHO], 2017). Dzau et al. (2020) discuss the risks of a parallel pandemic specific to mental health if organizations do not react quickly to protect their staff.

Longitudinal studies are consistent in showing that the COVID-19 pandemic exacerbated mental health problems (Daly et al., 2020; Pierce et al., 2020) and that these effects may even have been underestimated (Czeisler et al., 2021). This crisis context illustrates the extent to which organizations must strike a balance between productivity on the one hand and the health and wellbeing of their employees on the other hand. Psychosocial safety climate (PSC) theory highlights the implications of attaining a balance between productivity and mental health for organizations and their staff. Specifically, PSC refers to “shared perceptions regarding policies, practices, and procedures for the protection of worker psychological health and safety,” and PSC represents “the causes of the causes of work stress” (Dollard and Bakker, 2010, p. 579).

Many studies have demonstrated the precursor role of PSC for work design and employee health, e.g., the reduction of emotional exhaustion (Idris et al., 2011, Idris et al., 2014; Mansour and Tremblay, 2019), but few researchers have used this theory to understand the role of PSC in work performance. Idris et al. (2011) showed that PSC was directly and positively related to perceived performance. In addition, these authors highlighted that PSC positively influences job resources, e.g., managerial support, thus increasing engagement at work, which, in turn, enhances performance. Conversely, a better PSC was associated with reduced work demands, which, in turn, reduced the risk of burnout. In their study, both burnout and engagement were predictors of job performance.

As Ipsen et al. (2020) argue that “good health is good for business” (p. 1) and that there is a need to address mental health and performance at work simultaneously in research and organizations because these two issues are intrinsically interrelated (Nowrouzi-Kia et al., 2021). Moments of crisis, such as those triggered by the COVID-19 pandemic, cause an upsurge in mental health problems but also create transformational opportunities for individuals and organizations. One such opportunity is the phenomenon of post-traumatic growth (PTG), which is the set of positive changes following a traumatic event (Tedeschi and Calhoun, 1996, 2004b). Although little studied in the context of a crisis (Gori et al., 2021), this form of growth may have been experienced by some employees. The experience of contracting COVID-19 can be traumatic for some individuals, leading them to experience increased anxiety, distress, and depression (Masiero et al., 2020; Cohen and Nica, 2021). For others, however, the experience can also lead to lasting changes in the way they view the world, e.g., appreciating life more, changing their relationship to work, or altering their spiritual life (Nearchou and Douglas, 2021). These opportunities for PTG during a health crisis may, on the one hand, depend on individual characteristics such as resilience, hope, or beliefs (Nearchou and Douglas, 2021; Vazquez et al., 2021). On the other hand, they may also depend on organizational context because some studies suggest that PTG is more likely in a context in which mental health issues are prioritized and supported by top management (Wood et al., 2020).

The present study examines the mediating role of psychological distress and PTG in the relationship between PSC and job performance. More precisely, this research raises the following questions: (1) by putting in place the appropriate practices, policies, and procedures related to psychological health, especially during a health crisis, can organizations limit their employees’ psychological distress while helping them achieve PTG? (2) To what extent does PSC influence employees’ performance during a health crisis? (3) To what extent are psychological health indicators such as psychological distress and PTG explanatory mechanisms for the relationship between PSC and performance? To answer these questions, this study analyzes PSC, psychological distress, PTG, and perceived performance.

### Psychosocial safety climate

A good PSC is characterized by freedom from psychological and social risk or harm at the highest levels of the organization (Dollard and Bakker, 2010). Specifically, PSC includes four dimensions: (1) top management commitment, namely their support in the prevention of work-related ill-being through the implementation of useful and decisive actions; (2) priority given to PSC by senior management, which is reflected in the importance placed on the psychological health and safety of employees vs. production; (3) communication, which refers to the organization’s ability to listen, dialog, and take into account its members’ contributions to psychological health and safety; and (4) organizational participation, which entails the consultation of employees and unions on issues related to psychological health and safety (Dollard and Bakker, 2010).

As an organizational resource likely to influence the constraints (i.e., by requiring compensatory physical and/or psychological efforts in order to cope with the situation while achieving the objectives set) and resources (i.e., by reducing the intensity of the constraints and their deleterious effects on health while stimulating personal growth and development) of a job (Hakanen et al., 2006), PSC can be considered an extension of the Job Demands-Resources model (JD-R: Demerouti et al., 2001; Schaufeli and Bakker, 2004; Bakker and Demerouti, 2007). The JD-R model is based on two distinct psychological processes: the health impairment process, which assumes that constraints lead to various health problems, e.g., depression (Hakanen et al., 2008), and the motivational process, which argues that resources have motivational potential because they promote employee learning and development (Bakker and Demerouti, 2007). Dollard et al. (2019) assert that PSC mitigates health problems indirectly by reducing constraints and their effects and increases work commitment indirectly through resources. More concretely, in a weak PSC context, employees and their managers may have no internal mechanism, e.g., reporting procedures or a counseling unit, enabling them to report individual (e.g., chronic fatigue, stress, and risk of burnout) or collective (e.g., work overload and interpersonal conflicts) difficulties to management. A good PSC implies that the organization gives a high priority to the mental health of staff and managers and puts in place the necessary mechanisms to ensure managers have the resources needed to support their staff. A good PSC has been associated with better managerial practices because it promotes better mental health for managers (Biron et al., 2018; Parent-Lamarche and Biron, 2022). In the same vein, a multi-level study of healthcare workers during the pandemic showed that PSC promotes resilience through hope, as well as increasing the impact of supportive leadership on employee hope (Siami et al., 2022). In contrast, when PSC is low, the means available to employees to report their difficulties may be inadequate or non-existent. As a result, the work-related constraints to which they are exposed are more likely to persist over the long term, affecting their health and performance (Liu et al., 2020; Biron et al., 2021). Similar findings (Idris et al., 2015; Lee and Idris, 2017) confirmed that PSC acts as an antecedent to job demands and resources. By strengthening employees’ job resources, e.g., learning opportunities, PSC increases their interest in and enthusiasm for their work, i.e., work engagement, as well as their performance.

Despite its theoretical soundness, few studies have considered the mechanisms through which PSC influences work performance during a health crisis. Therefore, this study analyzes the effects of PSC on the psychological health (psychological distress and posttraumatic growth) and performance of employees during a health crisis.

### Psychological distress

Psychological distress is generally used as an early indicator of mental disorder (Kessler et al., 2003b). It is associated with various symptoms, such as cognitive impairments, irritability, depression, and anxiety (Ching et al., 2021). Previous studies indicate that high psychological demands, low work support, and low recognition for work efforts are strong predictors of psychological distress (Duchaine et al., 2017). Regarding the consequences of psychological distress, these include decreases in work productivity due to absenteeism (Duchaine et al., 2020) and presenteeism (Biron et al., 2021). For example, a study by Mirza et al. (2019) conducted in a sample of the oil and gas workers in Malaysia has shown that psychological distress mediates the relationship between PSC and safety behaviors in such a way that PSC reduced psychological distress, which, in turn, improved safety behaviors. Like Mirza et al. (2019), in this study, we suggest that a psychologically safe climate will help reduce distress, which, in turn, will improve work performance.

**Hypothesis 1**. Psychosocial safety climate is negatively related to psychological distress.

### Post-traumatic growth

The COVID-19 pandemic has engendered or reinforced work-related constraints, e.g., job uncertainty, such that the work environment may now pose new risks to workers’ psychological health (Zahiriharsini et al., 2022). It has consequently become essential to identify the organizational variables, e.g., PSC, related to both positive and negative outcomes for employees, specifically in times of a health crisis.

Introduced by Tedeschi and Calhoun, 1996, 2004a,b, the concept of PTG corresponds to the set of positive changes following a traumatic event. More precisely, it describes the process of individuals experiencing these changes in certain areas of their lives through the reevaluation of their worldview (Gori et al., 2021). Although PTG is considered a salutogenic concept (Hamama-Raz et al., 2020), Tedeschi and Calhoun (2004a) clarify that, while PTG occurs more frequently in the context of suffering and inner struggle, it can also emerge in the lives of individuals who have not experienced specific trauma (Tedeschi and Calhoun, 1996), particularly in occupational settings (Sattler et al., 2014). For example, Stanton et al. (2006), in their review of the literature on the subject, suggest that cancer patients can experience PTG by, among other things, seeking more social support or using positive and adapted coping strategies. Accordingly, the constraints associated with the pandemic situation, e.g., successive lockdowns, may have both traumatic and constructive consequences (for a narrative review on PTG in the workplace during COVID-19, see Finstad et al., 2021; Vazquez et al., 2021).

Tedeschi and Calhoun (1996, 2004b) identified five areas that are central to the concept of PTG: personal strength, new possibilities in life, relationships with others, appreciation of life, and spiritual change. First, people who experience an increase in personal strength feel that they can better handle everyday tasks and events that had been perceived as insurmountable, e.g., hard-to-achieve goals or internal conflicts. Second, PTG involves the identification of new possibilities for oneself and one’s life, such as taking a different path than one had planned, e.g., career reorientation or a change in career development) (Tedeschi and Calhoun, 2004b). Third, PTG is characterized by potentially more intimate interpersonal relationships. Individuals thus become more aware of the importance of their relationships and cherish them more. This change also results in increased compassion for others, e.g., during a restructuring or job loss (Tedeschi and Calhoun, 2004b). Fourth, greater appreciation of life can also qualify as a PTG experience. For example, many aspects of daily life, however, small, are associated with small joys that can take on special meaning. The sense of priorities is profoundly altered such that “little things” are more valued, e.g., time spent with loved ones (Tedeschi and Calhoun, 2004b). Finally, the PTG experience can include positive changes in spirituality. People who experience PTG, be they religious or not, often engage in spiritual and existential reflection, which helps them cope with painful emotions or loss (Tedeschi and Calhoun, 2004b). To summarize, the PTG experience allows individuals to engage in a cognitive process, e.g., positive reinterpretation, positive reframing, interpretive control, and the reconstruction of events, that imparts meaning to their experiences and future perspectives. It allows them to develop resources with which to cope with new and undesirable situations (Hobfoll, 2002; Sattler et al., 2014).

Post-traumatic growth is increasingly being investigated in work settings (e.g., physicians, Taku, 2014; firefighters, Yang and Ha, 2019; paramedics, Ragger et al., 2019), but occupational factors are rarely considered. The literature has focused on the benefits of individual (e.g., emotional intelligence, Li et al., 2015; optimism, Yang and Ha, 2019; sense of coherence, Ragger et al., 2019) or personal (e.g., family support, Taku, 2014) characteristics in terms of PTG; scant research has explored organizational avenues of action. However, a few studies have noted the positive influence of the meaning of work (Hamama-Raz et al., 2020), recognition at work (Idås et al., 2019), and perceived social support in the workplace (Sattler et al., 2014) on PTG. Maitlis (2020) endorses various organizational practices that promote the development of PTG in employees, such as establishing a supportive organizational culture for employees coping with trauma, paying special attention to teams that are suffering, and creating organizational conditions that promote interpersonal trust and psychological safety.

**Hypothesis 2**. Psychosocial safety climate is positively related to post-traumatic growth.

### Relationships between psychosocial safety climate, distress, growth, and performance

Organizational performance reflects a firm’s results, ranging from productivity to profitability, while remaining dependent on employees’ perceived performance (Ipsen et al., 2020). Depending on their efficiency levels, personnel may or may not achieve the objectives set by the employer. This is why many authors emphasize the fact that psychological health and performance are intrinsically linked (Peccei and Van De Voorde, 2019) so that employees with good psychological health report better performance than those with poor psychological health. Despite organizational and governmental policies that assume a lack of connection between health and performance (Hasle et al., 2019), Ipsen et al. (2020) argue that these variables should be examined and integrated jointly into central managerial concerns and practices. The present study attempts to respond to this call by focusing on workers’ psychological health and performance simultaneously.

Performance indicators vary widely between studies and can include subjective, e.g., perceived performance (Shimazu et al., 2010), or objective measures, e.g., total sales volume (Shannahan et al., 2013). It can be self-reported or not, e.g., completed by the supervisor (Alessandri et al., 2017), and can also be protean with respect to the profession studied, e.g., safe behavior among oil and gas workers (Mirza et al., 2019).

Kessler et al. (2003a) recommend examining performance as a subjective and global construct whereby employees evaluate their overall performance according to their own criteria. Although this operationalization does not allow one to distinguish among employees’ skills, behaviors, and results, it does allow one to put these factors into perspective and determine whether employees have met the organization’s requirements (Shimazu and Schaufeli, 2009; Shimazu et al., 2010). Moreover, this approach seems particularly well suited to a representative sample of a national population, as may be the case in our study, i.e., the French population.

One of the main objectives of examining performance is to identify the variables that best predict it, particularly during a health crisis in which labor shortages are acute. Thus, employees’ health is construed as a key determinant of performance (Ipsen et al., 2020), such that wellbeing and ill-being will have differentiated effects. For example, several studies have shown that sleep disorders (Giorgi et al., 2018), psychological ill-being (Huang and Simha, 2018), and perceived stress (Lindegård et al., 2014) lead to performance deterioration. In addition, a few studies find that psychological distress is negatively related to performance (Lim and Tai, 2014) because distress leads to decreased attention, motivation, and effort. Conversely, several studies demonstrate that engagement at work (Shimazu and Schaufeli, 2009) and subjective wellbeing (Salgado et al., 2019) increase performance. Similar results were also found during the COVID-19 pandemic. Nemteanu et al. (2021) showed that job satisfaction positively influenced performance, while negatively affecting counterproductive behaviors. Similarly, Prodanova and Kocarev (2021) highlighted the negative indirect influence of information and communications technologies (ICT) anxiety on work-from-home performance via job efficacy.

**FIGURE 1:**
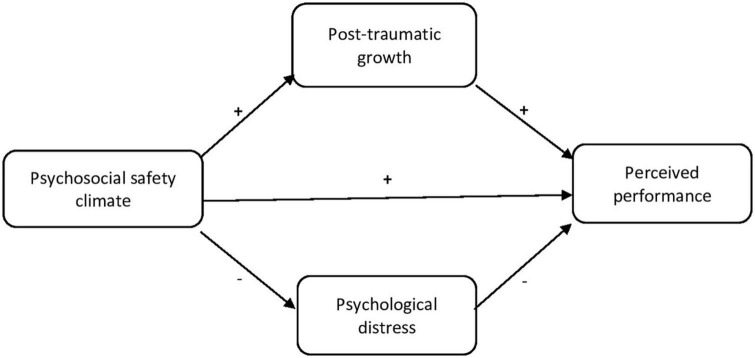
Theoretical model.

Although no research to date has examined the influence of PTG on performance, it is likely that, as a salutogenic indicator, the resources with which PTG is associated, e.g., improved self-image and higher quality of interpersonal relationships, allow employees to experience more positive effects and events perceived as stimulating and, thus, to adopt the appropriate behaviors so as to achieve high levels of performance. Thus, we offer the following hypotheses (Figure 1).

**Hypothesis 3**. Psychological distress is negatively related to perceived performance, whereas PTG is positively related to perceived performance.

**Hypothesis 4**. The positive relationship between PSC and perceived performance is mediated by psychological distress and post-traumatic growth.

## Materials and methods

### Participants and procedure

All participants in this study were recruited through a French opinion polling institute, OpinionWay, with which we collaborated in this work. The participants completed an online questionnaire between October 19 and 28, 2020. In an invitation was sent by email, in which they were told how to access the questionnaire. The targeted sample was representative of the characteristics of the working population in France, e.g., the ratio of men to women, and aged 18 years or more. The representativeness of the sample was based on quota methods for gender, age, and profession, which was performed after stratification by region and town size. In addition, the participants were told that this research was anonymous and confidential, that there were no right or wrong answers, and that it was important to answer sincerely. The survey was completed in no more than 20 min. The socio-demographic and socio-professional characteristics of the participants are presented in Table 1.

**TABLE 1:** Characteristics of study participants.

	Participants (*N* = 2,004)	Percentage
**Gender**		
Men	1,042	52.0
Women	962	48.0
**Age**		
18–29 years	183	9.1
30–39 years	581	29.0
40–49 years	625	31.2
50–59 years	493	24.6
60 years and older	122	6.1
**Seniority in the organization**		
Less than 1 year	142	7.1
1–3 years	373	18.6
3–5 years	332	16.5
5–9 years	262	13.1
10 years or more	895	44.7
**Job categories**		
Executives and professionals	583	29.1
Middle managers	476	23.8
Employees	682	34.0
Laborers	263	13.1
**Company size**		
Less than 10 employees	263	13.1
10–249 employees	746	37.2
250–4,999 employees	601	30.0
5,000 employees or more	394	19.7
**Supervision**		
Managers	685	34.2
Managers of managers	217	10.8
**Work status during the pandemic**		
Full time	1,743	87.0
Partial technical unemployment	171	8.5
Total technical unemployment	53	2.6
Leave/special leave of absence	37	1.9
**Days teleworked/week (*n* = 1,914)**		
No teleworking	1,236	64.6
1 day a week	107	5.6
2 days a week	224	11.7
3 days a week	170	8.9
4 days a week	60	3.1
5 days a week	110	5.7
6 days a week	4	0.2
7 days a week	3	0.2

## Measures

### Psychosocial safety climate

Participants reported their perceptions of their organization’s PSC based on four items (α = 0.90 for this study; i.e., “Senior management shows support for stress prevention through involvement and commitment,” “Senior management considers employee psychological health to be as important as productivity,” “There is good communication here about psychological safety issues which affect me,” “In my organization, the prevention of stress involves all levels of the organization” (Dollard, 2019). The instructions they were given took into account the COVID-19 pandemic context (i.e., “*The following statements relate to psychological health and safety within your organization. Considering your current employment status during this pandemic, please select the answer that best fits your situation*”). Responses ranged from 1 (Strongly Disagree) to 5 (Strongly Agree).

### Psychological distress

The six items of the Kessler Psychological Distress Scale (K6; Kessler et al., 2002, 2003b) were used to measure the frequency with which participants exhibited symptoms of non-specific psychological distress the week prior, e.g., feeling nervous, depressed, agitated, or irritable (α = 0.90 for this study). The response choices ranged from 1 (Never) to 5 (All the time). This measure was used because it reflects the diagnostic criteria for psychological unhappiness, specifically major depression and generalized anxiety disorder (Kessler et al., 2002). The K6 has been validated with adults in several studies; its psychometric properties are as good as those of the K10 (Kessler et al., 2002, 2003b; Furukawa et al., 2003). The scale can also be used with established threshold to discriminate cases of serious mental problems from non-cases (Kessler et al., 2003b,^2010^).

### Post-traumatic growth

The post-traumatic growth inventory (PTGI), developed by Tedeschi and Calhoun (1996), measures perceived benefits following a traumatic event. Participants responded to a total of 21 items. Specifically, they were asked to report the extent to which events related to the health context, i.e., the declaration of the COVID-19 pandemic, confinement, and re-opening, caused lasting changes (α = 0.95 for this study; “I have new interests,” “I feel closer to others,” “I have a greater appreciation of the value of my life”). The responses ranged from 1 (Not at all) to 5 (Totally).

### Perceived performance

Performance was measured by responses to the following question: “Over the past week, how would you rate your performance at work on a scale of 0–100%?” (Kessler et al., 2003a). The responses ranged from 0% (the worst performance an employee could deliver) to 100% (the best performance an employee could achieve) in 5% increments. The main reason for the choice of this scale is that the nature of the performance indicators varies significantly from one study to another. This makes it all the more difficult to examine work performance when it is studied in a population-based sample, such as the one used in this study. In this respect, some authors propose to measure performance through a one-item subjective scale (Shimazu and Schaufeli, 2009; Shimazu et al., 2010), which allows us to take into account disparate professional backgrounds.

## Analyses

The data were analyzed using the Statistical Package for Social Sciences (SPSS 23) software. To test the set of hypotheses, several steps were followed. First, descriptive and correlation analyses were conducted to explore the relationships between the variables, i.e., PSC, psychological distress, PTG, and perceived performance. Second, analyses were conducted to test the mediating effects of psychological distress and PTG on the relationship between PSC and perceived performance. To this end, the procedure defined by Hayes and Preacher (2014) was used. It involves estimating four parameters (i.e., alpha, which corresponds to the regression weight of PSC on each mediator, namely psychological distress and PTG; beta, which corresponds to the regression weight of the mediators of perceived performance; c, which corresponds to the total effect (i.e., direct and indirect) of PSC on perceived performance; and c′, which corresponds to the direct effect of PSC on perceived performance (indirect effect = c − c′). We can thus differentiate the direct and indirect effects of an independent variable on a dependent variable. Finally, the indirect effect is calculated as the product of the alpha × beta relationships for each mediator. Its 95% confidence interval is estimated from a resampling procedure that is repeated 5,000 times. This commonly used procedure produces a more reliable estimate of the confidence interval because it is robust to a non-normal distribution on the part of the indirect effect (Preacher and Hayes, 2008). These mediation analyses were conducted using the freely available macro PROCESS v3.5 (model 4) developed by Hayes (2022).

## Results

First, correlation analyses were performed to test for preliminary support for our hypotheses (see Table 2). Our results showed that PSC is positively correlated with PTG and perceived performance [*r*(2,003) = 0.24 and *r* = 0.21, respectively; *p* < 0.001] but negatively correlated with psychological distress [*r*(2,003) = −0.223; *p* < 0.001]. In addition, psychological distress and PTG are, respectively, negatively [*r*(2,003) = −0.21; *p* < 0.001] and non-significantly [*r*(2,003) = 0.033; *p* > 0.05] related to perceived performance.

**TABLE 2:** Means, standard deviations, and correlations.

	*M*	SD	Correlations			
			
Variables			1	2	3	4
(1) Psychosocial safety climate	2.96	1.01	–			
(2) Psychological distress	2.26	0.90	−0.223**	–		
(3) Post-traumatic growth	3.31	1.02	0.246**	−0.012	–	
(4) Perceived performance	78.55	21.84	0.219**	−0.377**	0.033	–

**p ≤ 0.01.

Next, simple mediation analyses were performed to identify potential mechanisms, i.e., psychological distress and PTG, via which PSC influences perceived performance. The results are presented in Table 3.

**TABLE 3:** Results of direct and indirect effects of mediation.

Direct effects					

	Mediators	*B*	ES	*t*	*p*
PSC-performance (total relationship)	–	4.68	0.46	10.02	<0.001
PSC-performance (direct relationship)	–	3.06	0.46	6.59	<0.001
PSC-mediators (alpha relationships)	PD	–0.19	0.02	–10.24	<0.001
	PTG	0.24	0.02	11.33	<0.001
Mediators-performance (beta relationships)	PD	–8.33	0.50	–16.40	<0.001
	PTG	–0.12	0.45	–0.27	0.78

**Indirect effects**					

	**Effects**	**Effects (%)**	**BootSE**	**BootLLCI**	**BootULCI**

PSC-PD-performance	1.65	35	0.20	1.26	2.06
PSC-PTG-performance	−0.03	0.6	0.11	–0.25	0.19

PSC, psychosocial safety climate; PD, psychological distress; PTG, post-traumatic growth.

Psychosocial safety climate is negatively associated with psychological distress (*b* = −0.19; CI = [−0.23; −0.16]; *p* < 0.001) but positively associated with PTG (*b* = 0.24; CI = [0.20; 0.29]; *p* < 0.001). Furthermore, results showed that psychological distress negatively predicted perceived performance (*b* = −8.33; CI = [−9.32; −7.33]; *p* < 0.001), whereas PTG was not significantly associated with perceived performance (*b* = −0.12; CI = [−1.00; 0.76]; *p* = 0.78). Consistent with these results, we found that PSC has an indirect and positive influence on perceived performance by reducing psychological distress (*b* = 1.64; CI = [1.26; 2.06]). Conversely, PTG did not make a significant indirect contribution to the relationship between PSC and perceived performance (*b* = −0.03; CI = [−0.25; 0.19]).

## Discussion

This study examined the effects of PSC on psychological distress, PTG, and perceived performance among French employees during the COVID-19 pandemic, specifically prior to the second confinement in France (October 30 to December 15, 2020). First, as hypothesized, our results indicate that PSC is positively related to PTG but negatively related to psychological distress. These results support H1 and H2. Our results partially supported H3, showing that distress was negatively associated with performance but the association with PTG was not significant. As for H4, the association between PSC and performance was partially mediated by psychological distress. PSC indirectly fostered work performance by reducing psychological distress. The mediating effect of PTG was not significant.

### Theoretical contributions

Our results are consistent with previous work that found that PSC was associated with positive consequences for both psychological health and performance (Idris et al., 2015). More tangibly, PSC is an organizational resource that tends to mitigate constraints such as work overload, whilst promoting resources such as social support, autonomy, and skills development (Dollard and Bakker, 2010; Yulita et al., 2022). PSC implies that key stakeholders are enabled to respond promptly and proactively to the psychological health issues exacerbated by the pandemic. PSC has been associated in previous studies with many mental health outcomes such as psychological distress (Platania et al., 2022; Yulita et al., 2022), and with the core components of the JD-R model such as burnout and engagement (Dollard and Bakker, 2010; Idris et al., 2011). However, to the best of our knowledge, it has never been used in the context of crisis as an antecedent to PTG, thus responding to the recent call to use PSC with a broader range of outcomes (Dollard et al., 2019). This implies that employees who evolve in a work climate in which they perceive that their wellbeing is considered and preserved by their organization report less psychological distress and tend to experience the COVID-19 crisis more positively. The PSC thus helps maintain healthy working conditions, allowing employees to thrive professionally through good health and strong performance.

Second, consistent with previous research (Shimazu et al., 2010; Lim and Tai, 2014), our results showed that psychological distress was negatively associated with perceived performance. Furthermore, we demonstrate that PSC positively influences perceived performance via psychological distress. In other words, psychological distress is an explanatory mechanism for the relationship between climate and performance such that, when the PSC is perceived to be high, performance levels increase via a decrease in distress levels. These results are coherent with the Conservation of Resources theory (Hobfoll, 1989, 2002). Hobfoll and Shirom (2000) postulate that, when individuals have the necessary resources, e.g., a strong PSC, to cope with the constraints of their environment, they are also able to conserve and renew individual resources to preserve their wellbeing. Employees with sufficient resource reservoirs can undertake various projects at the workplace, intellectual challenges, or new career or training opportunities because they have the energy and motivation to achieve these goals. Conversely, if employees lack the necessary resources, e.g., weak psychosocial security climate, to perform their work despite certain constraints, e.g., a lack of autonomy or recognition, they risk developing higher levels of ill-being, e.g., psychological distress, and being unable to achieve their performance objectives. For employees, high levels of psychological distress are often associated with lower levels of concentration (Lim and Tai, 2014) and work engagement (Inoue et al., 2010). They thus become inattentive and put forth less effort when carrying out their tasks.

Third, contrary to our expectations, our results suggest that PTG is not significantly correlated with perceived performance and that it does not mediate the relationship between climate and performance. Accordingly, although PSC promotes the development of PTG, which is beneficial to employees’ psychological health, it does not enhance workers’ performance. This can be explained mainly via conceptual reasons linked to the very definition of PTG and its components. Tedeschi and Calhoun (1996, 2004b) identified five factors that are central to the concept of PTG: personal strength, new possibilities in life, relationships with others, appreciation of life, and spiritual change. While it is true that this growth allows individuals to develop new resources through pleasurable emotional, interpersonal, or spiritual experiences, it is also possible that the benefits of these experiences remain highly personal. In other words, the benefits experienced through PTG do not induce changes or improvements in performance but, rather, in individual wellbeing. For example, although PTG does not influence employee performance directly, it remains associated with significant reservoirs of resources that employees can draw on. Since PSC was found to be a positive determinant of PTG, future research could investigate the explanatory mechanisms behind this association. For example, PTG may depend not only on contextual factors such as PCS, but also on leadership behaviors specific to PTG, as suggested by Wood et al. (2020) in their study of a military sample. Lastly, as pointed out by Maitlis (2020), it is likely that certain aspects of growth are not enacted behaviorally.

### Limitations and future research directions

Although this study deepens our understanding of the relationship between PSC and perceived performance during a health crisis through two indicators of psychological health (psychological distress and PTG), it has limitations that deserve mention. First, this work is based on a transverse study protocol, which does not allow us to demonstrate causal relationships between our constructs, e.g., PSC and PTG. Therefore, longitudinal and experimental studies should be conducted to confirm and generalize these results, both within a representative sample of the French population and with more specific professionals, e.g., teachers, or hierarchical levels, e.g., local managers.

Second, we examined the extent to which specific indicators of psychological health, i.e., psychological distress and PTG influence perceived employee performance. However, we did not consider any objective performance indicators that could limit social desirability bias, i.e., the tendency to distort self-descriptions in a positive sense (McCrae and Costa, 1983), nor did we consider multisource measures, e.g., co-workers and supervisors, that could minimize common variance bias, i.e., variance in the dimensions studied attributable to the measurement method rather than to the constructs that the measures are assumed to represent (Podsakoff et al., 2003). Although we used only tools whose psychometric qualities had been confirmed repeatedly, future research could draw on multi-source data, e.g., peer-perceived organizational citizenship behaviors, and other indicators of organizational health, e.g., absenteeism and turnover. Multi-item and multi-dimensional scales would also be welcome because, while this tool has advantages, e.g., the ability to survey a sample with a variety of jobs, it does not allow for the examination of specific behaviors associated with performance, e.g., organizational citizenship behavior, nor the achievement of more concrete organizational objectives, e.g., the quality of brand and product presentation, including those relating to the COVID-19 pandemic, such as performance while teleworking.

The present study was conducted in the context of a health crisis, but it would be relevant to contrast these results with data collected in a less turbulent context. For example, Dollard and Bailey (2021) showed that, in times of crisis, as well as in non-crisis times, PSC can be developed and sustained with leaders and teams through appropriate interventions. Placing mental health as a priority for top management is even more relevant given that the pandemic has generated and even exacerbated emerging risks, such as unethical culture, technological pressure, and the management of organizational change (Zahiriharsini et al., 2022). As suggested by Dollard and Bailey (2021), the pandemic has put mental health on the radar of policy makers. This has led to a multitude of interventions that are not always grounded in theory or empirical evidence. Our study corroborates previous ones highlighting the fact that the PSC is a key target for both mental health and organizational performance (Idris et al., 2015; Biron et al., 2021; Dollard and Bailey, 2021; Parent-Lamarche and Biron, 2022).

### Practical implications

Our results underline the benefits of PSC for employees’ psychological health and performance in the context of a health crisis, particularly during periods of confinement. Therefore, it is essential for organizations to put in place policies, practices, and procedures explicitly intended to preserve workers’ psychological health and safety (Dollard and Bakker, 2010; Dollard et al., 2019). These measures could include developing an internal process to encourage employees to share their problems during a health crisis, e.g., individual or group interviews on health and psychological safety, and proposing internal solutions to address them. For example, the health context has disrupted many work practices, e.g., the deployment of telecommuting, and compartmentalized departments and colleagues, leading to feelings of isolation. In cases in which difficulties regarding teleworking, e.g., work overload and an imbalance between life areas, reach top managers, it could be interesting to train all the staff in good practices related to telework in order to avoid an increase in working hours, i.e., starting earlier and finishing later, and mental overload related to household tasks, e.g., looking after the children while attending a meeting via videoconference. Concurrently, drawing on Dollard and Bailey (2021), managers could be trained in practices that take such difficulties into account, on the one hand, by equipping them to recognize the signals of ill-being in their teams and, on the other hand, by enabling them to address the associated emotional load.

## Conclusion

Overall, this research sheds light on the role of PSC in perceived performance via two distinct mental health pathways, namely psychological distress and PTG. This expands the scope of studies that have primarily considered the effects of PSC on mental health, thus attempting to answer the call of Ipsen et al. (2020) to consider mental health and performance simultaneously rather than separately, as is most often the case in research and practice. Given the deterioration of mental health in many workplaces as a result of the pandemic and critical and pervasive labor shortages in several work sectors, it is crucial that leaders develop better practices, policies, and procedures to ensure that workers can work in psychologically safe environments.

## Data availability statement

The raw data supporting the conclusions of this article will be made available by the authors, without undue reservation.

## Ethics statement

Ethical review and approval was not required for the study on human participants in accordance with the local legislation and institutional requirements. The patients/participants provided their written informed consent to participate in this study.

## Author contributions

ÉS, J-PB, and CN contributed to conception and design of the study. ÉS and CN organized the database. ÉS and HI performed the statistical analysis. ÉS and J-PB wrote the first draft of the manuscript. CB and HI wrote sections of the manuscript. All authors contributed to manuscript revision, read, and approved the submitted version.
